# Targeting TRPV1 for Body Weight Control using TRPV1^−/−^ Mice and Electroacupuncture

**DOI:** 10.1038/srep17366

**Published:** 2015-12-01

**Authors:** Monchanok Choowanthanapakorn, Kung-Wen Lu, Jun Yang, Ching-Liang Hsieh, Yi-Wen Lin

**Affiliations:** 1 College of Chinese Medicine, Graduate Institute of Acupuncture Science and International Master Program, China Medical University, Taichung 40402, Taiwan; 2 College of Chinese Medicine, School of Post-Baccalaureate Chinese Medicine, China Medical University, Taichung 40402, Taiwan; 3 China Medical University Hospital, Department of Chinese Medicine, Taichung, 40402, Taiwan; 4 College of Chinese Medicine, Graduate Institute of Integrative Medicine, China Medical University, Taichung 40402, Taiwan; 5 Research Center for Chinese Medicine & Acupuncture, China Medical University, Taichung 40402, Taiwan.

## Abstract

Obesity is a global social medical problem resulting in morbidity as high as 20–30%. Here we investigated whether the manipulation of TRPV1 can control mice body weight through electroacupuncture (EA). The results demonstrated that body weight increased with time in the control group (108.19 ± 1.31%, n = 7). The increase of mice body weight was significantly less in the EA group (104.41 ± 0.76%, p < 0.05, compared with the control group, n = 7) but not in the sham EA group (109.1 ± 0.63%, p < 0.05, compared with EA group, n = 7). EA did not decrease the gain of body weight in TRPV1 knock mice (107.94 ± 0.41% and 107.79 ± 1.04% for TRPV1^**−**/**−**^ and TRPV1^**−**/**−**^ with EA, respectively, p > 0.05). The visceral white adipose tissue (WAT) weight was lower in the EA group at 4 weeks after manipulation. Moreover, the protein levels of TRPV1, pPKA, pPKC, and pERK were increased in the dorsal root ganglion (DRG) and spinal cord (SC) after EA treatment but not in the sham EA and TRPV1^**−**/**−**^ mice. This study suggests that targeting TRPV1 is beneficial in controlling body weight and TRPV1-associated mechanisms in mice.

Obesity is a global social medical problem resulting in morbidity as high as 20–30%. In addition, the number of obese patients across the world is now on the rise along with the cost of health insurance^1^. Obesity is related to various chronic diseases and common particularly in areas where food supplies are plentiful and lifestyles are sedentary. The degree of obesity is generally described by the body mass index (BMI). Simple obesity is one of the most serious epidemics around the world and is receiving ever-increasing attention from the medical profession. At present, obesity has replaced malnutrition and infectious diseases and has become one of the most important human health hazards^2^. Obesity associated with insulin resistance is of increasing significance with aging because it leads to the development of type 2 diabetes mellitus. Moreover, it severely influences the physical and mental health, weakens immunity, and increases the mortality rate. Even excess weight has reached epidemic proportions globally, with more than 2 billion adults being either overweight or obese worldwide^3^.

TRPV1 is a heat/proton/lipid/voltage-modulated Ca^2+^ -permeant (PCa/PNa approximately 10) ion channel^4^. A more voltage-gating-centric explanation is that at warmer temperatures (>37 °C) or in the presence of capsaicin, TRPV1 current is activated by a high physiological range of voltages^5^,^6^. Endogenous cannabinoid receptor ligands, such as anandamide, are potential TRPV1 agonists. The size of its current is increased by acid pH and is modulated by intracellular PIP2, which inhibits the channel^7^. Experiments using TRPV1 knockout mice confirm that it is essential for transducing the nociceptive, inflammatory, and hypothermic effects of vanilloid compounds and contributes to acute thermal nociception and thermal hyperalgesia post tissue injury^8^,^9^. TRPV1 current is potentiated by bradykinin and nerve growth factor via several possible mechanisms, including PLC-mediated protein kinase C (PKC) activation and/or PIP2 hydrolysis and phosphatidylinositol 3-kinase^7^,^10^. It was recently shown that electroacupunture (EA) can relieve inflammatory and fibromyalgia pain through TRPV1^11^,^12^,^13^. Thus, emerging data indicate that TRPV1 antagonists could also be useful in treating disorders other than pain, such as urinary urge incontinence, chronic cough, and irritable bowel syndrome^14^. TRPV1 is expressed by a subset of small-sized dorsal root ganglion (DRG) or trigeminal ganglion neurons^15^,^16^. The expression of TRPV1 has been demonstrated in cutaneous sensory nerves, mast cells, and epithelial cells^17^. Group IV muscle afferents were activated by either acid or capsaicin^18^, and intra-arterial injection of capsazepine, which is an antagonist of TRPV1, attenuated the cardiovascular response evoked by the injection of acid into the hindlimb muscle^19^.

PKC has been identified as an important signal transduction molecule that regulates various proteins involved in the regulation and maintenance of myocardial function. PKC exists as a family of isoforms, including the conventional PKCs (α, β1, β2, and γ), novel PKCs (δ, ε, η, and θ), and atypical PKCs (λ and ζ)^20^. PKC plays a major role in transmembrane signal transduction^21^. The activation of PKCε, as well as membrane targeting and substrate specificity, is regulated by several factors, including phosphorylation, diacylglycerol (DAG), and other lipids and anchoring proteins. However, several studies have consistently identified that the activation of PKCε plays a central role in the signaling pathways and cellular events that provide myocardial protection from ischemia and/or reperfusion injury^22^,^23^,^24^.

Extracellular signal-regulated kinase (ERK1/2) promotes cellular proliferation in response to growth factors. ERK signaling also regulates the expression of autophagy and lysosomal genes^25^. The activation of the ERK signaling pathway in the periphery is likely necessary for the maintenance of a spinally sensitized state, while the activation of ERK1/2 in the primary injury site may regulate TRPV1, leading to dorsal horn hypersensitivity to thermal and chemical stimuli^26^. The activation of ERK in primary afferent neurons is mediated, at least partly, by TRPV1^27^. The auricular EA was recently shown to reduce epilepsy accompanied by altered pERK signaling pathway in rats^28^.

Acupuncture originated in ancient China at least 2,500 years ago. While treating obesity using acupuncture, it is essential to select the points based on differentiation of the symptoms and signs, and multiple points in multiple meridians should be selected. The expression of TRPV1 in nerve fibers is significantly increased by EA stimulation in acupoints; moreover, the higher expression of TRPV1 in the subepidermal nerve fibers and its upregulation after EA stimulation may play a key role in mediating the transduction of EA signals to the central nervous system (CNS), and its expression in the subepidermal connective tissue cells may play a role in conducting the local effect of EA^29^. Acupuncture is used for treating various health problems, one of which is obesity^30^ and EA reduces body weight in overweight subjects in clinical practice, as well as in rats and mice with diet-induced obesity^31^.

EA is highly reported to reduce body weight in obese subjects in clinical practice; the phenomenon was also observed in rats and mice with high fat diet-induced obesity. In the current study, we hypothesized that EA activates the peripheral nervous system to transfer signals to CNS for controlling body weight. Our results revealed that body weight was controlled by using TRPV1^**−**/**−**^ mice or EA-treated mice. The white adipose tissue (WAT) weight was also lower in TRPV1^**−**/**−**^ mice or EA-treated mice. The mechanism leading to these results was through the activation of TRPV1-related signaling pathway such as pPKA, pPKC, and pERK.

## Materials and Methods

### Experimental Animals

Adult male C57/B6 (BioLASCO Taiwan Co., Ltd) mice aged 8 to 12 weeks were used in this study. The usage of these animals was approved by the *Institute of Animal Care and Use Committee of China Medical University (permit No. 101-116-N), Taiwan. All experiments were performed in accordance with the use of Laboratory Animals* (National Academy Press). We use EA on mice by inserting a stainless steel acupuncture needles (1.5” inch, 32G, YU KUANG, Taiwan) into the ST36 acupoint at a depth of 3–4 mm. Square pulses electrical stimulation were delivered for 15 min with a duration of 100 μs and 2 Hz in frequency generated from the stimulator. The stimulation amplitude was 1 mA. The similar protocol was given to gluteal muscle non acupoint to set as the sham EA group. Mice were subdivided into 5 groups (1) Control group (2) EA group (3) sham-EA group (4) TRPV1^**−**/**−**^ mice (5) TRPV1^**−**/**−**^ mice with EA. Each group had 7 mice. Mice in all groups received sufficient food and water ad libitum. The weight of mice and the amount of food and water consumed were monitored. The number of animals used and their suffering was minimized.

### Western blot analysis

L3-L5 DRG and lumbar SC neurons were immediately excised to extract protein at week 4 and stored at –80 °C. Total proteins were prepared by homogenized sample in lysis buffer containing 50 mM Tris-HCl pH 7.4, 250 mM NaCl, 1% NP-40, 5 mM EDTA, 50 mM NaF, 1 mM Na3VO4, 0.02% NaN3 and 1 × protease inhibitor cocktail (AMRESCO). The extracted proteins (30 μg per sample assessed by BCA protein assay) were subjected to 8% SDS-Tris glycine gel electrophoresis and transferred to a PVDF membrane. The membrane was blocked with 5% nonfat milk in TBS-T buffer (10 mM Tris pH 7.5, 100 mM NaCl, 0.1% Tween 20), incubated with anti-TRPV1, anti-pPKA, anti-pPKC, anti-pERK, anti-pp38, and anti-pJNK antibody (1:1000, Alomone) in TBS-T with 1% bovine serum albumin, and incubated for 1 hour at room temperature. Peroxidase-conjugated anti-rabbit antibody (1:5000) was used as a secondary antibody. The bands were visualized by an enhanced chemiluminescencent substrate kit (PIERCE) with LAS-3000 Fujifilm (Fuji Photo Film Co. Ltd). Where applicable, the image intensities of specific bands were quantified with NIH ImageJ software (Bethesda, MD, USA).

### Data Analysis

All statistic data were presented as the mean ± standard error. Statistical comparisons were performed with One Way ANOVA test, followed by a post hoc Tukey’s test (*p* < 0.05 was considered statistically significant).

## Results

### Effects of EA at ST36 acupoint on reducing body weight WAT gain in mice

**Figure 1:**
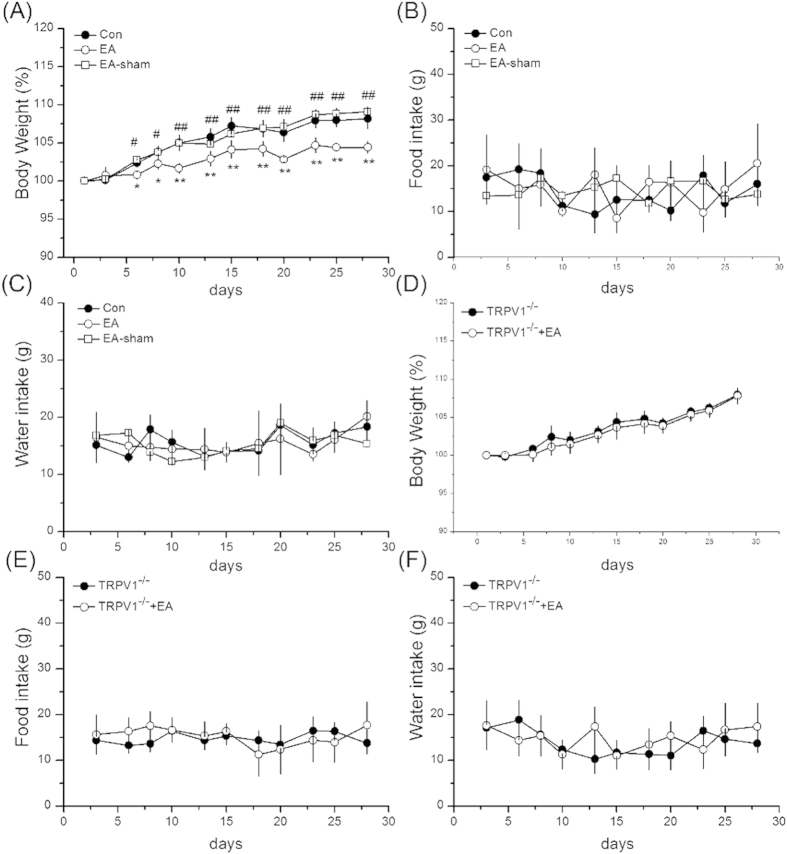
Electroacupuncture (EA) at ST36 acupoint attenuated body weight gain but not food and water intake. (**A**) Body weight gain in control, EA and sham groups. (**B**) Food intake in control, EA and sham groups. (**C**) Water intake in control, EA and sham groups. (**D**) Body weight gain in TRPV1^**−**/**−**^ and TRPV1^**−**/**−**^ + EA groups. (**E**) Food intake in TRPV1^**−**/**−**^ and TRPV1^**−**/**−**^ + EA groups. (**E**) Water intake in TRPV1^**−**/**−**^ and TRPV1^**−**/**−**^ + EA groups. Con = control; EA = electroacupuncture; sham = sham-EA group. *p < 0.05 compared to baseline; **p < 0.01 compared to baseline; ^#^*p* < 0.05 compared to baseline ^##^*p* < 0.01 compared to baseline (n = 7 mice per group).

We investigated whether EA at ST36 acupoint can reduce body weight gain. In the control group, the body weights were increased with time (Fig. 1, 108.19 ± 1.31%, *p* < 0.05, compared with baseline, n = 7). We further performed EA on normal mice, and the results indicated that EA could reliably reduce the weight gain (Fig. 1, 104.41 ± 0.76% *p* < 0.05, compared with control group, n = 7). Similar results were not observed if electrical stimulation was employed in non-acupoint gluteal maximus muscle (GM) area supporting an acupoint specificity (Fig. 1, 109.1 ± 0.63%, *p* < 0.05, compared with the EA group, n = 7). Further, we measured the body weight using TRPV1 knockout mice, which showed a similar increase in body weight as that of the control mice (Fig. 1, 107.94 ± 0.41%, *p* < 0.05, compared with baseline, n = 7). Importantly, EA cannot reduce body weight gain in TRPV1^**−**/**−**^ mice suggesting a crucial target for EA manipulation (Fig. 1, 107.79 ± 1.04%, *p* < 0.05, compared with TRPV1^**−**/**−**^ mice, n = 7).

**Figure 2:**
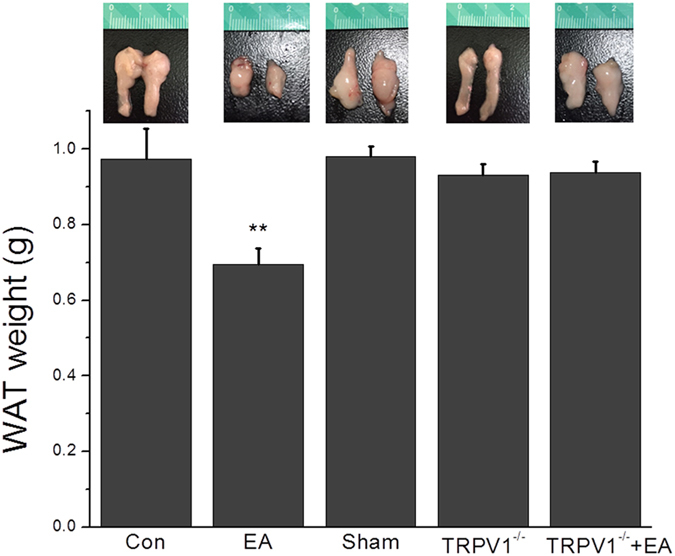
Electroacupuncture (EA) at ST36 acupoint reduced WAT weight in mice. WAT weight in Con, EA, sham, TRPV1^**−**/**−**^ and TRPV1^**−**/**−**^ + EA mice. Con = control; EA = electroacupuncture; sham = sham-EA group **p < 0.01, compared to baseline (n = 7 mice per group).

We then assessed if EA at ST36 acupoint can decrease the body weight by attenuating WAT weight. In the control mice, the visceral WAT weighed 0.97 ± 0.08 g (Fig. 2, n = 7). The WAT weight decreased after 4 weeks of EA stimulation at ST36 acupoint (Fig. 2, 0.69 ± 0.04 g, *p* < 0.05, compared with the control group, n = 7). This pattern was not obtained in mice receiving sham-EA manipulation (Fig. 2, 0.98 ± 0.02 g, *p* < 0.05, compared with the EA group, n = 7). The WAT weights were similar to those of the control group in both TRPV1^**−**/**−**^ and TRPV1^**−**/**−**^ mice with EA (Fig. 2, 0.93 ± 0.03 g and 0.94 ± 0.03 g, *p* < 0.05, compared with the EA group, n = 7). Because food and water intake are crucial for body weight control, we further checked if these were affected by EA. Both the food and water intake of mice in all the groups were similar (Fig. 1, n = 7, *p* > 0.05). These results showed that EA could ameliorate the increase of body weight by reducing the WAT tissue weight rather than food and water intake.

### Quantification of TRPV1 and related signaling molecules in DRG

**Figure 3:**
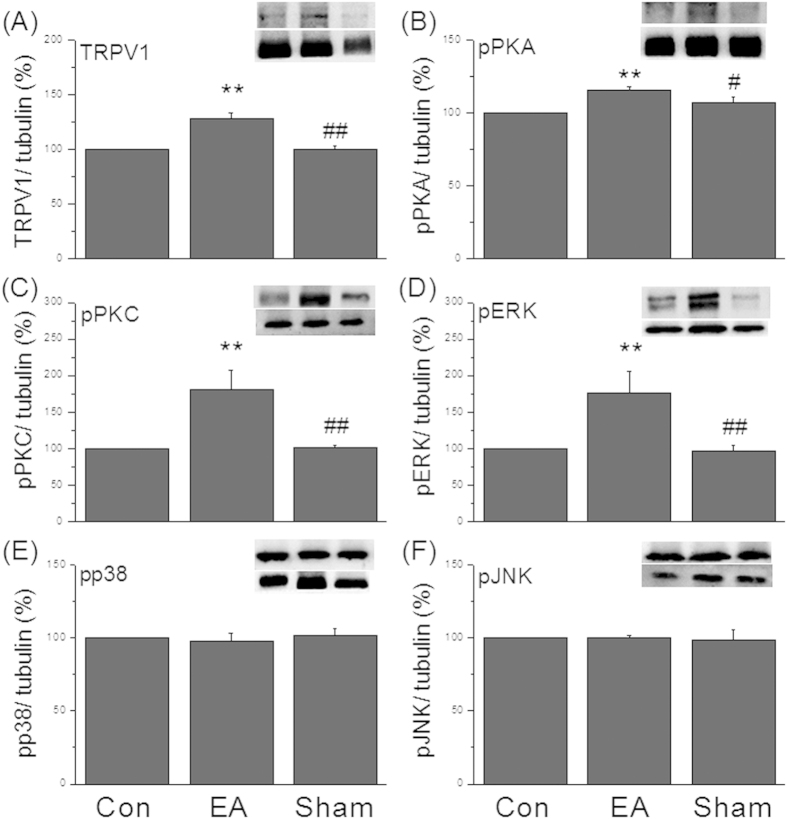
Upregulation of TRPV1 and related signal mechanisms in DRG neurons from EA but not sham mice. (**A**) Western blot staining showed TRPV1-positive band; (**B**) pPKA-positive band; (**C**) pPKC-positive band; (**D**) pERK-positive band; (**E**) pp38-positive band; (**F**) pJNK-positive band in Con, EA, sham groups. Con = control; EA = electroacupuncture; sham = sham-EA group.

We tested the effects of EA at ST36 acupoint on the expression of TRPV1 in DRG through western blotting. The expression level of TRPV1 was normal in the control mice (Fig. 3, 100%, n = 7). The results showed that the protein level of TRPV1 was significantly increased after EA treatment at week 4 (Fig. 3, 128.47 ± 5.19%, p < 0.05, compared with the control group, n = 7). The phenomenon was not observed in sham-EA mice (Fig. 3, 100.1 ± 3.21%, p < 0.05, compared with the EA group, n = 7). Because TRPV1 is a Ca^2+^ permeable ion channel, which can trigger second messenger molecules, we further examined if EA can activate TRPV1 and the related mechanisms. It was observed that the protein level of pPKA was increased in the EA-treated mice (Fig. 3, 116.1 ± 2.44%, *p* < 0.05, compared to the control group, n = 7) but not in the sham-EA group (Fig. 3, 107.47 ± 9.68%, p < 0.05, compared to the control group, n = 7). Similar results were also obtained in the pPKC protein level suggesting its role in EA-mediated mechanisms (Fig. 3, 180.35 ± 26.48% and 100.95 ± 3.66% for EA and sham EA, respectively, *p* < 0.05, n = 7). We further assessed if pPKA and pPKC could trigger MAPK family. The results revealed that the protein level of pERK was significantly increased after EA treatment (Fig. 3, 175.91 ± 30.59%, *p* < 0.05, compared to the control group, n = 7) but not in the sham control mice (Fig. 3, 98.98 ± 7.03%, p < 0.05, compared to the EA group, n = 7). These results were not observed in other members of the MAPK family such as pp38 (Fig. 3, 97.73 ± 5.95% and 97.73 ± 6.77% for EA and sham-EA group, respectively, *p* < 0.05, n = 7) and pJNK, indicating that pERK is a key element in EA manipulation (Fig. 3, 100.29 ± 2.06% and 101.88 ± 4.71% for EA and sham-EA group, respectively, *p* < 0.05, n = 7).

### Identification of TRPV1 and the related mechanisms in spinal cord (SC)

**Figure 4:**
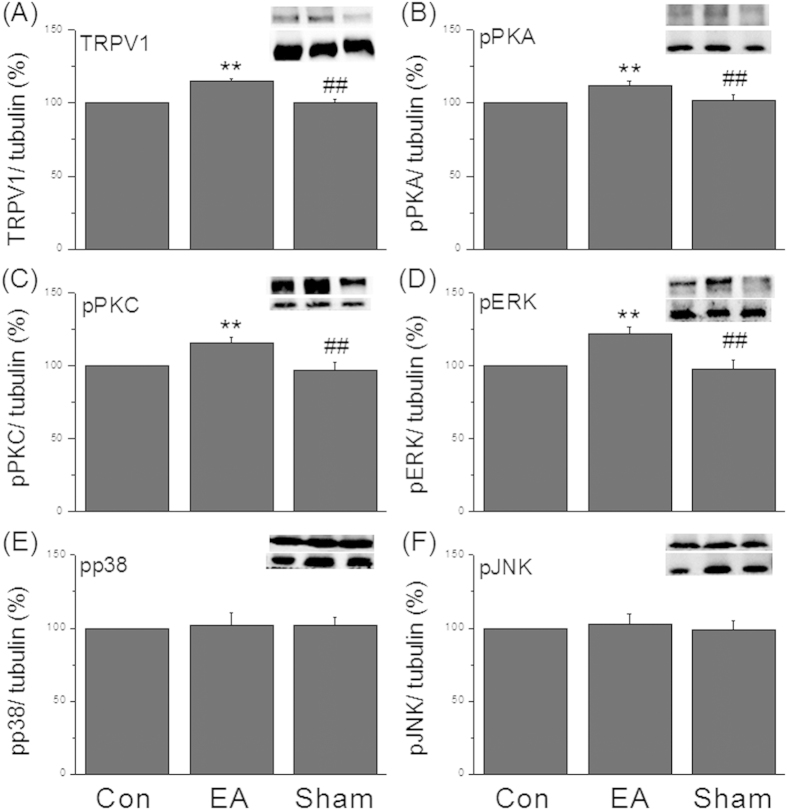
Upregulation of TRPV1 and relevant molecules in SC from EA but not sham-EA mice. (**A**) Western blot staining showed TRPV1-positive band; (**B**) pPKA-positive band; (**C**) pPKC-positive band; (**D**) pERK-positive band; (**E**) pp38-positive band; (**F**) pJNK-positive band in Con, EA, sham groups. Con = control; EA = electroacupuncture; sham = sham-EA group.

To verify if EA can regulate body weight at central–spinal level, we tested TRPV1 and the related downstream mechanisms using SC sample. TRPV1 was increased after EA treatment (Fig. 4, 115.45 ± 1.43%, p < 0.05, compared to the control group, n = 7) but not in the sham-EA group (Fig. 4, 100.8 ± 2.97%, p < 0.05, compared to the EA group, n = 7). Our results showed that the quantity of pPKA was increased after EA manipulation (Fig. 4, 112.87 ± 2.68%, *p* < 0.05, compared to the control group, n = 7) but not in the sham-EA group (Fig. 4, 108.17 ± 10.62%, p < 0.05, compared to the control group, n = 7). Similar data were also observed in pPKC in the EA-treated group (Fig. 4, 102.94 ± 5.21% and 96.69 ± 5.3% for EA and sham-EA group, respectively, *p* < 0.05, n = 7). These results also showed that pERK was drastically increased after EA treatment (Fig. 4, 121.87 ± 4.62%, *p* < 0.05, compared to the control group, n = 7) but not in the sham control (Fig. 4, 98.88 ± 6.04%, p < 0.05, compared to the EA group, n = 7). The results were not obtained in pp38 (Fig. 4, 101.74 ± 8.59% and 97.79 ± 6.45% for EA and sham-EA group, respectively, *p* < 0.05, n = 7) and pJNK (Fig. 4, 102.95 ± 6.35% and 102.26 ± 5.27% for EA and sham-EA group, respectively, *p* < 0.05, n = 7).

### Characterization of TRPV1-associated pathways in DRG and SC using TRPV1^−/−^ mice

**Figure 5:**
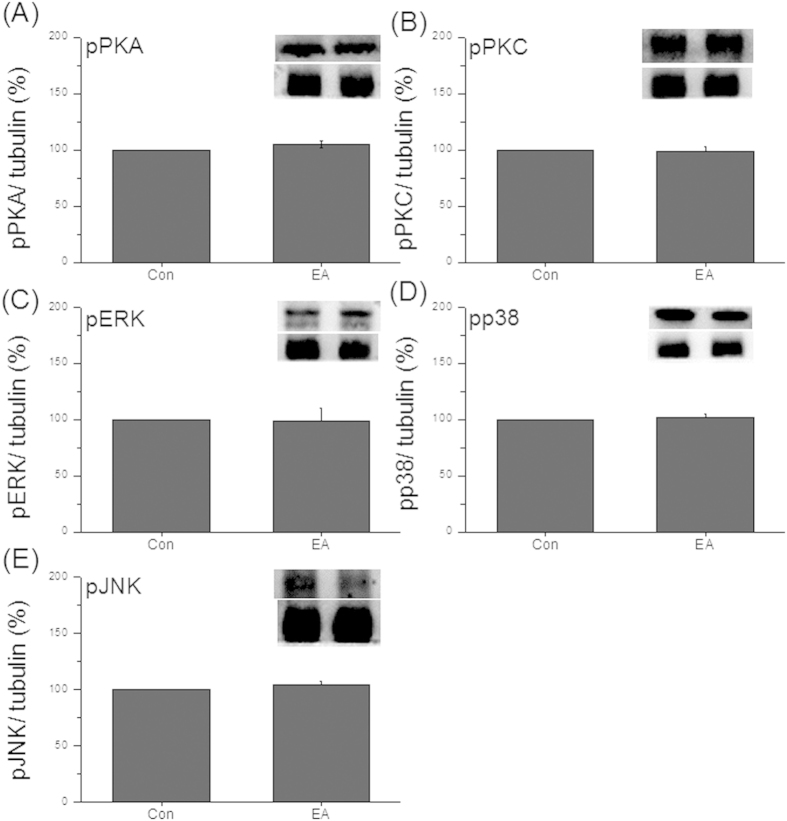
Protein levels of TRPV1-related signal mechanisms were unchanged in DRG neurons in TRPV1^**−**/**−**^ mice mice. (**A**) Western blot staining showed pPKA-positive band; (**B**) pPKC-positive band; (**C**) pERK-positive band; (**D**) pp38-positive band; (**E**) pJNK-positive band in in TRPV1^**−**/**−**^ and TRPV1^**−**/**−**^ + EA groups.

**Figure 6:**
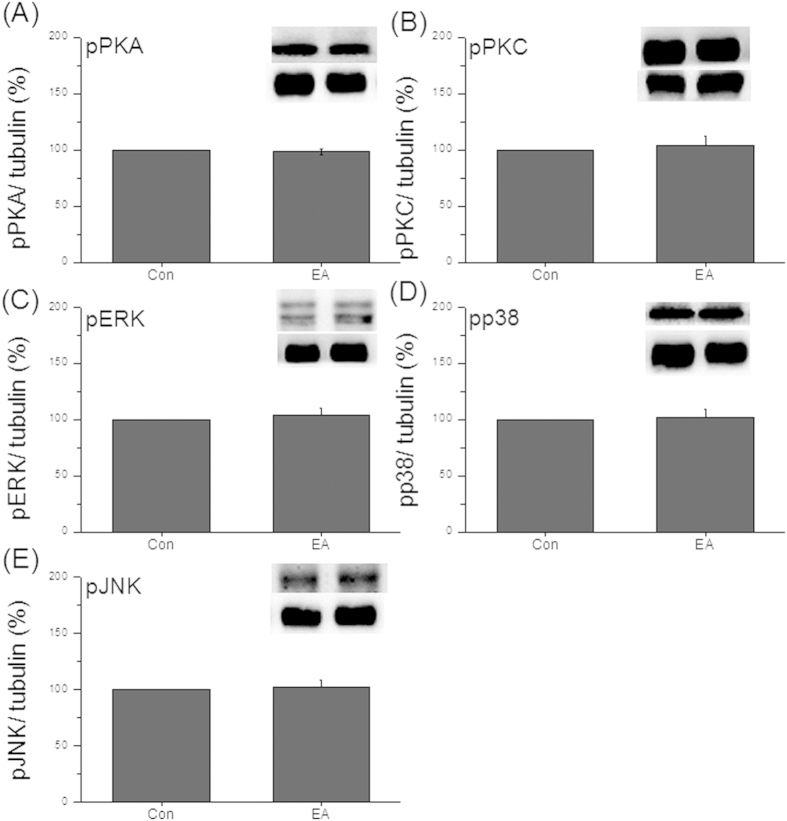
Protein levels of TRPV1-associated molecules were unaltered in SC neurons from TRPV1^**−**/**−**^ mice. (**A**) Western blot staining showed pPKA-positive band; (**B**) pPKC-positive band; (**C**) pERK-positive band; (**D**) pp38-positive band; (**E**) pJNK-positive band in TRPV1^**−**/**−**^ and TRPV1^**−**/**−**^ + EA groups.

As the results indicated that EA couldn’t regulate body weight in TRPV1^**−**/**−**^ mice, we next assessed if EA can increase TRPV1-mediated signaling pathways. It was observed that pPKA was unchanged in DRG after EA stimulation at week 4 (Fig. 5, 104.61 ± 3.41%, *p* > 0.05, compared to the TRPV1^**−**/**−**^ group, n = 7). Similar data were also obtained in the pPKC level (Fig. 5, 99.1 ± 4.06%, *p* > 0.05, compared to the TRPV1^**−**/**−**^ group, n = 7). Furthermore, our results also showed that pERK was not altered after EA treatment (Fig. 5, 98.93 ± 11.48%, *p* > 0.05, compared to the TRPV1^**−**/**−**^ group, n = 7). The same results were also observed in pp38 (Fig. 5, 102.39 ± 3.02%, *p* > 0.05, compared to the TRPV1^**−**/**−**^ group, n = 7) and pJNK (Fig. 5, 103.85 ± 3.45%, *p* > 0.05, compared to the TRPV1^**−**/**−**^ group, n = 7). Moreover, pPKA was unaltered in SC after 4 weeks of EA stimulation (Fig. 6, 98.29 ± 2.62%, *p* > 0.05, compared to the TRPV1^**−**/**−**^ group, n = 7). The protein level of pPKC was similar in TRPV1^**−**/**−**^ mice and TRPV1^**−**/**−**^ mice with EA treatment (Fig. 6, 104.29 ± 7.44%, *p* > 0.05, compared to the TRPV1^**−**/**−**^ group, n = 7). Altogether, the results were similar in pERK (Fig. 6, 103.89 ± 6.53%, *p* > 0.05, compared to the TRPV1^**−**/**−**^ group, n = 7), pp38 (Fig. 6, 101.96 ± 6.89%, *p* > 0.05, compared to the TRPV1^**−**/**−**^ group, n = 7), and pJNK (Fig. 6, 102.08 ± 5.92%, *p* > 0.05, compared to the TRPV1^**−**/**−**^ group, n = 7). These results indicated that EA could not regulate the body weight in mice lacking TRPV1.

## Discussion

In the current study, we showed that the gain of body weight was increased with time in the control group. The gain of body weight was lower in the EA-treated mice than in the sham control. Furthermore, EA cannot decrease the gain of body weight in TRPV1^**−**/**−**^ mice. The food and water consumption was similar in all the groups for the 4 weeks of study period. The weight of visceral WAT was lower after EA treatment. Furthermore, the protein levels of TRPV1, pPKA, pPKC, and pERK were increased in DRG and SC after EA treatment. Our results suggest that targeting TRPV1 is beneficial in controlling body weight through pPKA, pPKC, and pERK pathways.

Obesity is a component of the metabolic syndrome that is often associated with an increased risk of coronary heart disease, type II diabetes, stroke, osteoarthritis, and certain types of cancer. These deleterious conditions are detrimental to the individuals’ quality of life and cause emotional and financial burden on the individuals, their families, and the society as a whole^32^. Acupuncture has been shown to have beneficial effects on obesity. Several studies also showed that laser acupuncture has beneficial effects in treating obesity^33^.

Although the mechanisms of acupuncture for treating obesity remain unclear, it involves a suppression of appetite^34^; regulation of obesity-related peptides (increases cocaine and amphetamine-regulated transcript peptide and decreases ghrelin and leptin)^35^,^36^ and fat metabolism (decreases total cholesterol, triglycerides, low-density lipoprotein, lipoprotein A, and apolipoprotein B levels)^37^,^38^; and increases in insulin and C-peptide levels and, in cases of obesity, decreases in glucose levels^39^. Moreover, acupuncture is positively effective in treating obesity with its flexible point selection and various methods without toxic side effects^40^.

Yu *et al.* showed that the body weight of hot pepper–fed rabbits was drastically decreased compared with the control group suggesting that the intake of capsaicin prevents diet-induced obesity^41^. Our data are consistent with this issue that targeting TRPV1 is a potential for treating obesity. A recent study showed that rodents fed a diet containing 0.014% capsaicin indicated normal caloric intake but 24% reduction in the visceral fat. It was also suggested that these mechanisms were due to increased TRPV1 mRNA expression in visceral adipose tissue^42^. Ji *et al.* reported that body weights were decreased by EA stimulation in obese-prone (OP) rats. They also showed that TRPV1 mRNA was significantly increased in the nucleus tractus solitarius (NTS) after EA treatment in OP rats^43^. Shen *et al.* suggest that EA can remodel WAT into BAT by inducing UCP1 expression that may be one of the mechanisms by which acupuncture affects weight loss^44^. In the present study, we showed that TRPV1 was increased in both peripheral DRG and central SC.

## Conclusion

In summary, we speculate that EA at ST36 acupoint may be beneficial in reducing body weight with decreased WAT weight and increased TRPV1, pPKA, pPKC, and pERK expression. These findings suggest that TRPV1 is expressed and plays a role in the metabolic mechanisms and that EA is effective in reducing body weight. These findings can also be applied to further clinical studies.

## Additional Information

**How to cite this article**: Choowanthanapakorn, M. *et al.* Targeting TRPV1 for Body Weight Control using TRPV1^–/–^ Mice and Electroacupuncture. *Sci. Rep.* **5**, 17366; doi: 10.1038/srep17366 (2015).

## References

[b1] Bei Y., Fang X. & Yao Z. Sixty-two cases of simple obesity treated by acupuncture combined with massage. J. Tradit. Chin. Med. 24, 36–39 (2004). 15119170

[b2] Yang L. *et al.* Obesity and influenza associated mortality: evidence from an elderly cohort in Hong Kong. Prev. medicine. 56, 118–123 (2013). 10.1016/j.ypmed.2012.11.017 23219760

[b3] Ng M. *et al.* Global, regional, and national prevalence of overweight and obesity in children and adults during 1980-2013: a systematic analysis for the Global Burden of Disease Study 2013. Lancet 384, 766–781 (2014). 24880830 10.1016/S0140-6736(14)60460-8 PMC4624264

[b4] Caterina M. J. & Julius D. The vanilloid receptor: a molecular gateway to the pain pathway. Annu. Rev. Neurosci. 24, 487–517 (2001). 11283319 10.1146/annurev.neuro.24.1.487

[b5] Brauchi S., Orio P. & Latorre R. Clues to understanding cold sensation: thermodynamics and electrophysiological analysis of the cold receptor TRPM8. Proc. Natl. Acad. Sci. USA 101, 15494–15499 (2004). 15492228 10.1073/pnas.0406773101 PMC523461

[b6] Voets T. *et al.* The principle of temperature-dependent gating in cold- and heat-sensitive TRP channels. Nature 430, 748–754 (2004). 15306801 10.1038/nature02732

[b7] Chuang H. H. *et al.* Bradykinin and nerve growth factor release the capsaicin receptor from PtdIns(4,5)P2-mediated inhibition. Nature 411, 957–962 (2001). 11418861 10.1038/35082088

[b8] Caterina M. J. *et al.* Impaired nociception and pain sensation in mice lacking the capsaicin receptor. Science 288, 306–313 (2000). 10764638 10.1126/science.288.5464.306

[b9] Davis J. B. *et al.* Vanilloid receptor-1 is essential for inflammatory thermal hyperalgesia. Nature 405, 183–187 (2000). 10821274 10.1038/35012076

[b10] Premkumar L. S. & Ahern G. P. Induction of vanilloid receptor channel activity by protein kinase C. Nature 408, 985–990 (2000). 11140687 10.1038/35050121

[b11] Chen W. H. *et al.* Attenuation of TRPV1 and TRPV4 Expression and Function in Mouse Inflammatory Pain Models Using Electroacupuncture. Evid. Based. Complement. Alternat. Med. 2012, 636848 (2012). 23258994 10.1155/2012/636848 PMC3520481

[b12] Wu S. Y., Chen W. H., Hsieh C. L. & Lin Y. W. Abundant expression and functional participation of TRPV1 at Zusanli acupoint (ST36) in mice: mechanosensitive TRPV1 as an “acupuncture-responding channel”. BMC Complement. Altern. Med. 14, 96 (2014). 24612851 10.1186/1472-6882-14-96 PMC3984709

[b13] Lin J. G., Hsieh C. L. & Lin Y. W. Analgesic Effect of Electroacupuncture in a Mouse Fibromyalgia Model: Roles of TRPV1, TRPV4, and pERK. PLoS One 10, e0128037 (2015). 26043006 10.1371/journal.pone.0128037 PMC4456150

[b14] Szallasi A., Cortright D. N., Blum C. A. & Eid S. R. The vanilloid receptor TRPV1: 10 years from channel cloning to antagonist proof-of-concept. Nat. Rev. Drug. Discov. 6, 357–372 (2007). 17464295 10.1038/nrd2280

[b15] Jordt S. E. *et al.* Mustard oils and cannabinoids excite sensory nerve fibres through the TRP channel ANKTM1. Nature 427, 260–265 (2004). 14712238 10.1038/nature02282

[b16] Kobayashi K. *et al.* Distinct expression of TRPM8, TRPA1, and TRPV1 mRNAs in rat primary afferent neurons with adelta/c-fibers and colocalization with trk receptors. J. Comp. Neurol. 493, 596–606 (2005). 16304633 10.1002/cne.20794

[b17] Stander S. *et al.* Expression of vanilloid receptor subtype 1 in cutaneous sensory nerve fibers, mast cells, and epithelial cells of appendage structures. Exp. Dermatol. 13, 129–139 (2004). 14987252 10.1111/j.0906-6705.2004.0178.x

[b18] Hoheisel U., Reinohl J., Unger T. & Mense S. Acidic pH and capsaicin activate mechanosensitive group IV muscle receptors in the rat. Pain 110, 149–157 (2004). 15275762 10.1016/j.pain.2004.03.043

[b19] Gao Z., Henig O., Kehoe V., Sinoway L. I. & Li J. Vanilloid type 1 receptor and the acid-sensing ion channel mediate acid phosphate activation of muscle afferent nerves in rats. J. Appl. Physiol. 100, 421–426 (2006). 16210435 10.1152/japplphysiol.00659.2005

[b20] Nishizuka Y. The molecular heterogeneity of protein kinase C and its implications for cellular regulation. Nature 334, 661–665 (1988). 3045562 10.1038/334661a0

[b21] Nishizuka Y. Studies and perspectives of protein kinase C. Science 233, 305–312 (1986). 3014651 10.1126/science.3014651

[b22] Ping P. *et al.* Formation of protein kinase C(epsilon)-Lck signaling modules confers cardioprotection. J. Clin. Invest. 109, 499–507 (2002). 11854322 10.1172/JCI13200 PMC150872

[b23] Vondriska T. M. *et al.* Protein kinase C epsilon-Src modules direct signal transduction in nitric oxide-induced cardioprotection: complex formation as a means for cardioprotective signaling. Circ. Res. 88, 1306–1313 (2001). 11420308 10.1161/hh1201.092994

[b24] Ping P., Zhang J., Pierce W. M. Jr. & Bolli R. Functional proteomic analysis of protein kinase C epsilon signaling complexes in the normal heart and during cardioprotection. Circ. Res. 88, 59–62 (2001). 11139474 10.1161/01.res.88.1.59

[b25] Settembre C. *et al.* TFEB links autophagy to lysosomal biogenesis. Science 332, 1429–1433 (2011). 21617040 10.1126/science.1204592 PMC3638014

[b26] Li M. M. *et al.* Extracellular signal-regulated kinases mediate melittin-induced hypersensitivity of spinal neurons to chemical and thermal but not mechanical stimuli. Brain. Res. Bull. 77, 227–232 (2008). 18725270 10.1016/j.brainresbull.2008.07.009

[b27] Chen Y., Willcockson H. H. & Valtschanoff J. G. Vanilloid receptor TRPV1-mediated phosphorylation of ERK in murine adjuvant arthritis. Osteoarthritis Cartilage 17, 244–251 (2009). 18684647 10.1016/j.joca.2008.06.015 PMC2673950

[b28] Lin Y. W. & Hsieh C. L. Auricular electroacupuncture reduced inflammation-related epilepsy accompanied by altered TRPA1, pPKCα, pPKCε, and pERk1/2 signaling pathways in kainic acid-treated rats. Mediators of inflammation 2014, 493480 (2014). 25147437 10.1155/2014/493480 PMC4131505

[b29] Abraham T. S., Chen M. L. & Ma S. X. TRPV1 expression in acupuncture points: response to electroacupuncture stimulation. J. Chem. Neuroanat. 41, 129–136 (2011). 21256210 10.1016/j.jchemneu.2011.01.001 PMC3117662

[b30] He J. *et al.* Effect of combined manual acupuncture and massage on body weight and body mass index reduction in obese and overweight women: a randomized, short-term clinical trial. J. Acupunct. Meridian Stud. 8, 61–65 (2015). 25952121 10.1016/j.jams.2014.08.001

[b31] Tian N. *et al.* Electroacupuncture suppresses expression of gastric ghrelin and hypothalamic NPY in chronic food restricted rats. Peptides 27, 2313–2320 (2006). 16644064 10.1016/j.peptides.2006.03.010

[b32] Puhl R. M. & Heuer C. A. Obesity stigma: important considerations for public health. Am. J. Public. Health. 100, 1019–1028 (2010). 20075322 10.2105/AJPH.2009.159491 PMC2866597

[b33] Tseng C. C., Tseng A. & Chang C. H. Effect of laser acupuncture on obesity: study protocol for a randomized controlled trial. Trials 16, 217 (2015). 25972018 10.1186/s13063-015-0748-4 PMC4440285

[b34] Wang S. J., Yang H. Y. & Xu G. S. Acupuncture alleviates colorectal hypersensitivity and correlates with the regulatory mechanism of TrpV1 and p-ERK. Evid. Based Complement. Alternat. Med. 2012, 483123 (2012). 23097675 10.1155/2012/483123 PMC3477568

[b35] Tian D. R. *et al.* Up-regulation of the expression of cocaine and amphetamine-regulated transcript peptide by electroacupuncture in the arcuate nucleus of diet-induced obese rats. Neurosci. Lett. 383, 17–21 (2005). 15885905 10.1016/j.neulet.2005.03.039

[b36] Kim S. K. *et al.* The association of serum leptin with the reduction of food intake and body weight during electroacupuncture in rats. Pharmacol. Biochem. Behav. 83, 145–149 (2006). 16497365 10.1016/j.pbb.2006.01.002

[b37] Cabioglu M. T. & Ergene N. Electroacupuncture therapy for weight loss reduces serum total cholesterol, triglycerides, and LDL cholesterol levels in obese women. Am. J. Chin. Med. 33, 525–533 (2005). 16173527 10.1142/S0192415X05003132

[b38] Cabioglu M. T., Gundogan N. & Ergene N. The efficacy of electroacupuncture therapy for weight loss changes plasma lipoprotein A, apolipoprotein A and apolipoprotein B levels in obese women. Am. J. Chin. Med. 36, 1029–1039 (2008). 19051333 10.1142/S0192415X08006430

[b39] Cabioglu M. T. & Ergene N. Changes in levels of serum insulin, C-Peptide and glucose after electroacupuncture and diet therapy in obese women. Am. J. Chin. Med. 34, 367–376 (2006). 16710886 10.1142/S0192415X06003904

[b40] Zhang X. A clinical survey of acupuncture slimming. J. Tradit. Chin. Med. 28, 139–147 (2008). 18652123 10.1016/s0254-6272(08)60033-3

[b41] Yu Q. *et al.* Expression of TRPV1 in rabbits and consuming hot pepper affects its body weight. Mol. Biol. Rep. 39, 7583–7589 (2012). 22327653 10.1007/s11033-012-1592-1

[b42] Leung F. W. Capsaicin-sensitive intestinal mucosal afferent mechanism and body fat distribution. Life. Sci. 83, 1–5 (2008). 18541272 10.1016/j.lfs.2008.04.018

[b43] Ji B., Hu J. & Ma S. Effects of electroacupuncture Zusanli (ST36) on food intake and expression of POMC and TRPV1 through afferents-medulla pathway in obese prone rats. Peptides 40, 188–194 (2013). 23116614 10.1016/j.peptides.2012.10.009 PMC3646998

[b44] Shen W. *et al.* Acupuncture promotes white adipose tissue browning by inducing UCP1 expression on DIO mice. BMC. Complement. Altern. Med. 14, 501 (2014). 25514854 10.1186/1472-6882-14-501 PMC4301852

